# Multimethod feasibility evaluation of smoking cessation intervention for patients receiving opioid agonist therapy

**DOI:** 10.1186/s40814-025-01717-2

**Published:** 2025-10-31

**Authors:** Karl Trygve Druckrey-Fiskaaen, Tesfaye Madebo, Siv-Elin Leirvåg Carlsen, Einar Furulund, Jørn Henrik Vold, Jan Tore Daltveit, Torgeir Gilje Lid, Lars T. Fadnes, ATLAS4LAR Study Group, Karl Trygve Druckrey-Fiskaaen, Tesfaye Madebo, Siv-Elin Leirvåg Carlsen, Einar Furulund, Jørn Henrik Vold, Torgeir Gilje Lid, Lars T. Fadnes, Vibeke Bråthen Buljovcic, Trude Fondenes, Beate Haga Trettenes, Marianne Cook Pierron, Christine Sundal, Maren Borsheim Bergsaker, Eivin Dahl, Tone Lise Eielsen, Torhild Fiskå, Marianne Larssen, Eirik Holder, Ewa Joanna Wilk, Mari Thoresen Soot

**Affiliations:** 1 Department of Addiction Medicine, Bergen Addiction Research, Haukeland University Hospital, Bergen, Norway; 2 Department of Global Public Health and Primary Care, University of Bergen, Bergen, Norway; 3 Department of Clinical Sciences, University of Bergen, Bergen, Norway; 4 Department of Respiratory Medicine, Stavanger University Hospital, Stavanger, Norway; 5 Centre for Alcohol and Drug Research, Stavanger University Hospital, Stavanger, Norway; 6 Oral Health Centre of Expertise Rogaland, Stavanger, Norway; 7 Division of Psychiatry, Haukeland University Hospital, Bergen, Norway; 8 Faculty of Health Sciences, University of Stavanger, Stavanger, Norway

**Keywords:** Smoking cessation, Opioid agonist treatment, Opioid use disorder, Feasibility trial, Multimethod, Tobacco

## Abstract

**Background:**

Among patients receiving opioid agonist therapy, 85% are active tobacco smokers. Despite the significant impact of smoking-related pulmonary diseases on disability and mortality in this population, less than 10% successfully quit smoking with current interventions. The study aimed to evaluate the feasibility of integrating a smoking cessation intervention at outpatient clinics for persons receiving opioid agonist treatment. The specific outcomes were the feasibility of integration, intervention acceptability, participant retention, and the trial's impact on smoking behaviour.

**Methods:**

We conducted a 12-week feasibility study among patients receiving OAT (*n* = 25) in 2021 in Bergen, Norway. The feasibility outcomes were evaluated using qualitative semi-structured interviews and quantitative data collected at study visits during the pilot intervention period.

**Results:**

Participants reported that intervention was acceptable as it was easily accessible and provided for free at their local OAT clinic. Participants attended a median of 9 out of 12 weekly study visits and 40% of the participants completed all 12 weeks. The median number of cigarettes was reduced from 70 (range 31–175) to 26 (range 6–50) during the intervention period for those who attended all 12 study visits. One participant reported quitting smoking. The participants stressed that the possibility to individually choose smoking medication and tapering regimen was important for the acceptability of the intervention.

**Conclusions:**

Participant retention and qualitative results support the feasibility of the tested intervention, indicating that implementing integrated smoking cessation treatments in opioid agonist therapy clinics was feasible and acceptable to patients. The low number of participants quitting indicated low feasibility for smoking cessation. Simultaneously, the more than halving of the number of cigarettes smoked suggests that the progression of the intervention should have a reduction in the number of cigarettes smoked as the primary aim. However, the impact of this intervention requires thorough evaluation through a sufficiently powered randomised controlled trial.

**Supplementary Information:**

The online version contains supplementary material available at 10.1186/s40814-025-01717-2.

## Key messages regarding feasibility

- What uncertainties existed regarding the feasibility?
  There is uncertainty about whether a smoking cessation intervention can be integrated into the daily program at outpatient opioid agonist therapy clinics. Further, more information is needed on whether patients receiving opioid agonist therapy in outpatient clinics will experience a smoking cessation intervention as an integrated part of follow-up. Will the patients perceive the intervention as acceptable? Do patients manage to reduce smoking?
- What are the key feasibility findings?
  Our study indicated that the intervention could be integrated into the regular programme of the OAT clinics. Most participants managed to reduce smoking, but few stopped smoking. Participants valued the option to select both the smoking cessation product and the duration of dosage tapering steps. However, they desired increased flexibility in the intervention duration.
- What are the implications of the feasibility findings for the design of the main study?
  The participants should be allowed an extension of the intervention period with a longer duration of smoking cessation products. The intervention should involve all clinical staff providing follow-up with opioid agonist therapy at outpatient clinics. During the main trial, clinical staff will need structured training and follow-up.

## Introduction

Opioid dependence is a significant public health concern and is associated with a substantially increased risk of premature mortality and morbidity [1]. Approximately 85% of individuals receiving opioid agonist treatment (OAT) are tobacco smokers [2]; thus, smoking-related pulmonary diseases are substantial contributors to disability and mortality in this population [3, 4]. Despite this, the smoking cessation rates at 6–12 months among individuals receiving OAT, estimated at 6–10%, falls considerably below the rates observed in the general population [5, 6]. Lifetime quit ratios of 10–18% have been reported among individuals with opioid misuse or opioid use disorder [6]. Several factors contribute to this disparity, including interactions between opioids and nicotine, a societal and clinical environment that fosters smoking, the use of tobacco smoking as a coping mechanism for stress, and low expectations of smoking cessation among healthcare providers [7–9]. It is imperative to develop tailored interventions aimed at enhancing smoking cessation rates among individuals receiving OAT to address these multifaceted challenges.

Previous attempts at medically assisted smoking cessation using nicotine replacement products, varenicline or bupropion have demonstrated limited success within this population [9]. Smoking cessation is typically not sustained after the active intervention period [9–11]. Direct observed therapy with varenicline did not provide higher abstinence rates compared to self-administration of the medication [11]. Challenges such as the cost of medications and treatment adherence negatively impacted outcomes [12]. Behavioural treatments alone did not promote smoking cessation [9]. Contingency management strategies, such as monetary rewards for nicotine abstinence, have been shown to have some effect on smoking cessation [9, 13]. A recent trial in a similar population using a combination of motivational aspects and nicotine replacement products, showed a substantial reduction in smoking in the intervention group [14]. Qualitative research indicates that persons receiving OAT who smoke are concerned about the negative health effects of smoking, have a desire to quit, and have made previous quit attempts [15, 16]. The current study thoroughly examines these challenges, seeking to identify strategies that can mitigate cost-related barriers and provide support in reducing smoking.

The study aimed to evaluate the feasibility of integrating a combined smoking cessation intervention (smoking cessation medication and brief behavioural therapy) within the routine operations of OAT clinics, intending to reduce cigarette consumption. Additionally, it aimed to identify factors that could aid individuals undergoing OAT in reducing or quitting smoking. The goal was to devise effective strategies that address the unique challenges associated with smoking and achieving long-term cessation in this population.

## Method

### Study design

This multimethod study is a part of the ATLAS4LAR studies in Bergen and Stavanger, Norway [17, 18]. We used semi-structured individual interviews and quantitative data to evaluate the feasibility of the integrated smoking cessation intervention. The data was collected from May to October 2021 in OAT outpatient clinics in Bergen, Norway [17]. We used the CONSORT extension for randomised pilot and feasibility trials to guide the reporting of this study [19]. However, our study was not randomised, and certain checklist criteria were not applied (Additional File 1). The Consolidated criteria for reporting qualitative research (COREQ) were also used to guide the reporting (Additional File 2).

### Outcomes

The trial's outcomes were the feasibility of integration, intervention acceptability, participant retention, and the trial's impact on smoking behaviour. The outcomes were assessed using qualitative and quantitative data. See Table 1 for further details.

**Table 1:** Progression criteria

Progression criteria	Assessment
Feasibility of integration Can the smoking cessation intervention be provided alongside the ordinary OAT programme?	Qualitative interviews with participants Meetings with research nurses and research nurse field notes
Intervention acceptability Is the intervention acceptable to the participants?	Qualitative interviews with participants Overview of smoking cessation medication chosen Reported reasons for discontinuation
Feasibility of participant retention Will participants attend weekly study visits?	Assessing/counting weekly attendance per participant Qualitative interviews
The potential impact of the trial on smoking behaviour Does the trial have the potential to change self-reported smoking behaviour?	Qualitative interview Self-reported smoking intensity at the end of the trial

### Progression criteria

The progression criteria were agreed upon following several discussions in the research group, guided by the literature and the research group members' clinical experience.

### Sample size

The recommendations in the CONSORT statement [19] and the concept of information power [20] guided the sample size estimation. Research nurses purposely recruited participants whom they judged would be able to complete the study. Second, we aimed to reflect the age and sex distribution of the Norwegian OAT population (mean age of 47 years, with one-third females) as reported in [21]. Participants were recruited from several OAT clinics to ensure diversity and reduce the therapist effect [22]. In qualitative feasibility studies, study sizes usually range between 5 and 20 participants [22, 23]. After discussions in the research group, we determined that a sample size of 25 participants was adequate to ensure a balance between resources available, information expected to be obtained, and sufficient variability in the participant characteristics while at the same time allowing for drop-out, which is common in studies among persons with substance dependencies [24].

### Intervention

The intervention combined smoking cessation medication and brief behavioural treatment. All participants received a startup consultation with a research nurse to receive information about the study's aims and outcomes (smoking cessation or reduction). The participants were informed about the smoking cessation products. In the general population, meta-analyses have shown that combination therapy with long-acting (patches) and short-acting (lozenges or chewing gum) nicotine replacement therapy (NRT) had the highest chances of successful smoking cessation. When comparing single-form NRT treatment, short-acting NRT had similar success rates to nicotine patches [25]. In studies among persons with substance dependencies, pharmacotherapy alone or in combination with counselling, but not counselling alone, increased tobacco abstinence significantly [26]. Among persons with opioid dependency, smoking cessation medication showed lower efficacy than among persons without opioid dependence [9]. However, both varenicline and NRT were found to have modest effects on smoking cessation [9]. Based on the studies mentioned above indicating similar efficacy for long and short-acting NRT and varenicline, the participants were asked to choose a primary product, according to their preferences, among the following alternatives: NRT provided as patches (21 mg/24 h, 14 mg/24 h or 7 mg/24 h), lozenges or chewing gum (both in 1 mg or 2 mg per unit), or varenicline tablets (0.5 and 1 mg tablets). If the participants chose nicotine patches or varenicline tablets, they were allowed to use nicotine chewing gum or lozenges as additional as-needed medication. The dosing of the medication followed the manufacturers' instructions. Finally, the participants were given advice and a short motivational talk on how to stop or reduce smoking. During the intervention period, the participants were offered weekly study visits with a research nurse who provided a brief intervention on smoking cessation and discussed medication to be supplied for the next week. In addition, participants were asked weekly to estimate the daily number of cigarettes smoked during the past week and to rate the progression of the intervention from 1–5 (1 = very good, 2 = good, 3 = moderate, 4 = poor and 5 = very poor). Participants were allowed to change the smoking cessation medication, or the dosage used at each weekly consultation during the intervention period.

### Participants and recruitment

We recruited a purposive sample of 25 patients from five of the seven collaborating OAT clinics. Enrolment was halted when the pre-specified sample size was reached. Patients were eligible for treatment at the OAT clinics if they met the criteria for opioid dependency according to the International Classification of Diseases 10th revision and were at least 18 years old [27]. The OAT outpatient clinics are staffed with a multidisciplinary team of nurses, social workers, consultants in addiction medicine, and psychologists. In the clinics, patients receive their OAT medication under direct observation at least weekly. Additionally, they have the option to receive psychosocial and psychological treatments.

**Fig. 1:**
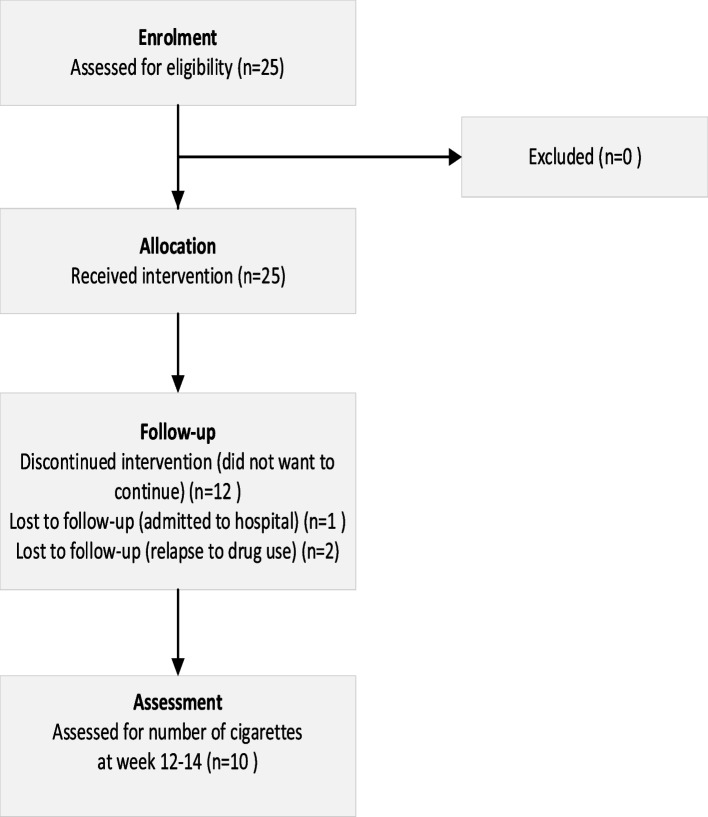
Flow chart of enrolment, follow-up, and assessment

Figure 1 shows the enrolment and follow-up, including the reasons for the discontinuation of the study. Research nurses at the OAT clinics recruited the participants. The inclusion criteria were as follows: 1) completed at least one health assessment with spirometry and self-reported daily cigarette use during the past week; 2) receiving OAT medication at least weekly; 3) smoked at least one cigarette per day during the past week; and 4) signed a written informed consent to participate in the trial. We excluded patients who 1) reported allergies or prior anaphylactic reactions to the intervention medications used, 2) smoked less than three days per week during the past week, or 3) were already using smoking cessation products. Participants were given a small incentive (equivalent of approximately 20 Euros) to compensate for the time used for participation*.*

### Data collection

Research nurses at the OAT clinics collected the quantitative data at baseline and during follow-up. Data on demographics, OAT medication, spirometry, education, living conditions, and debt was collected from the medical records at baseline. Data on cigarette use during the past week, as well as carbon monoxide in the exhaled air, and psychological well-being (assessed with the Norwegian validated translation ten-item version of Hopkins Symptom Checklist (SCL-10)) [28] was collected at baseline and the end of the trial (weeks 12–15) using electronic data collection software (CheckWare, CheckWare AS, Norway). Nicotine dependency (Fagerström) was only assessed at baseline [29]. The data was stored electronically in datasheets on a secure, access-restricted research server provided by Haukeland University Hospital. At weekly study visits, data on the number of cigarettes smoked during the last week was recorded, and measurements of carbon monoxide in the exhaled air were completed. Twenty-four patients were offered to participate in individual semi-structured interviews evaluating their experiences when completing the intervention. The English translation of the interview guide is provided in Additional file 3. The first author arranged the interviews with the patients at the OAT clinics, where they received regular follow-ups. The participants were informed that the interviews would be audio-recorded. The broad topics were their initial expectations for the intervention, motivation for participating, primary experience after study completion, perceived usefulness of medication for smoking cessation, and how the study affected smoking habits and lifestyle changes in general.

### Data analysis

#### Quantitative data

Clinical and demographic data was summarised to construct frequency and percentage tables and graphical presentations using Stata/SE17 (StataCorp, TX, USA, RRID: SCR_012763). Only data from participants (*n* = 10) who had completed the 12 weeks of the trial was included in the estimation and graphical presentation of changes in cigarette use.

### Qualitative data

We transcribed the interviews verbatim using NVivo software version 20 (RRID: SCR_014802). We used systematic text condensation to analyse the interviews. This systematic, step-by-step approach is suitable for thematic cross-case analyses [30, 31]. The analysis consisted of four main steps (Additional File 4). All authors except Daltveit and Vold, were involved in the qualitative analysis. The authors read all the transcripts, proposed preliminary themes, and discussed the preliminary themes, codes and quotes reflecting the codes during online video conferences with the other authors. First, the authors proposed themes that were discussed and refined until consensus was reached. Second, the researchers re-read the transcripts to identify meaning units, which were coded to the preliminary themes. Inter-coder reliability was ensured as coding of meaning units from the interviews onto the themes was subject to discussion among the authors. Based on these discussions, the first author wrote the condensation of the meaning units and the first draft of the final analytical text. A previous study described the analysis process in depth [15].

## Results

**Fig. 2:**
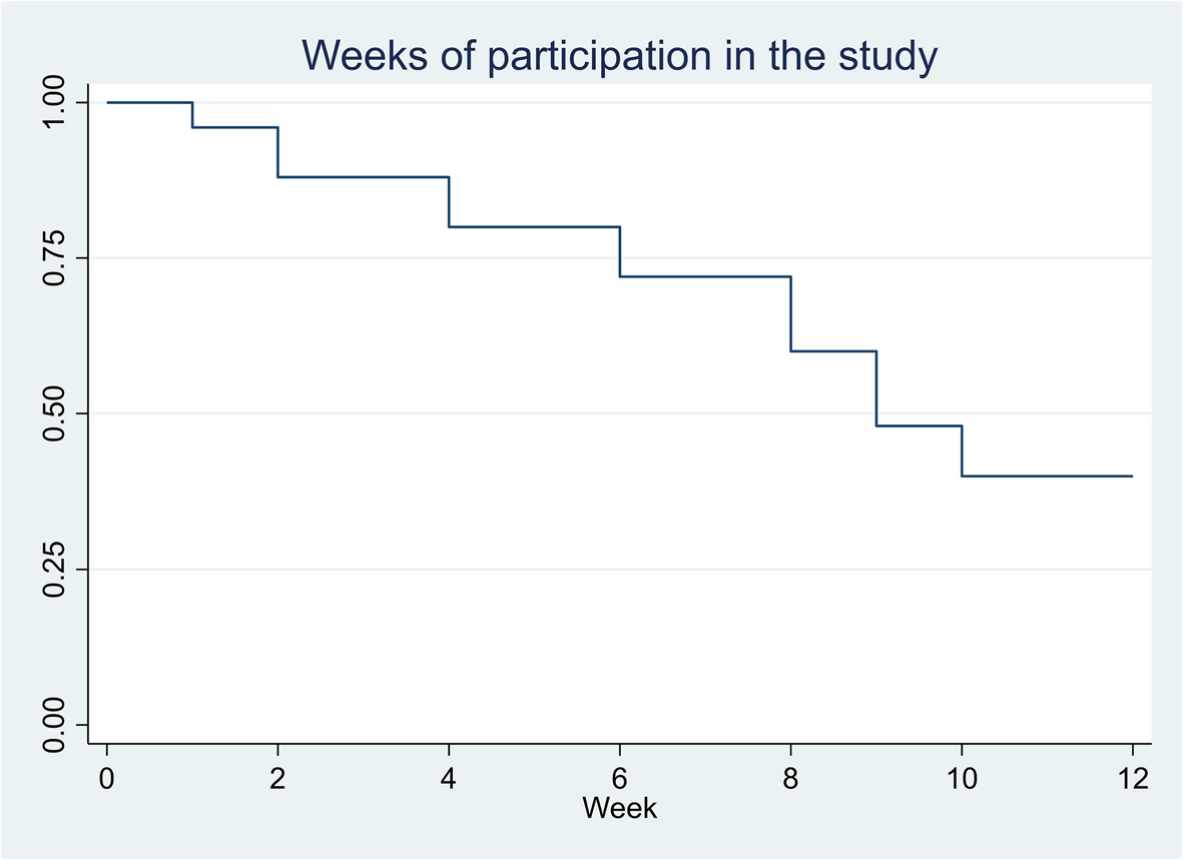
Weeks of attending study visits (*n* = 25)

**Fig. 3:**
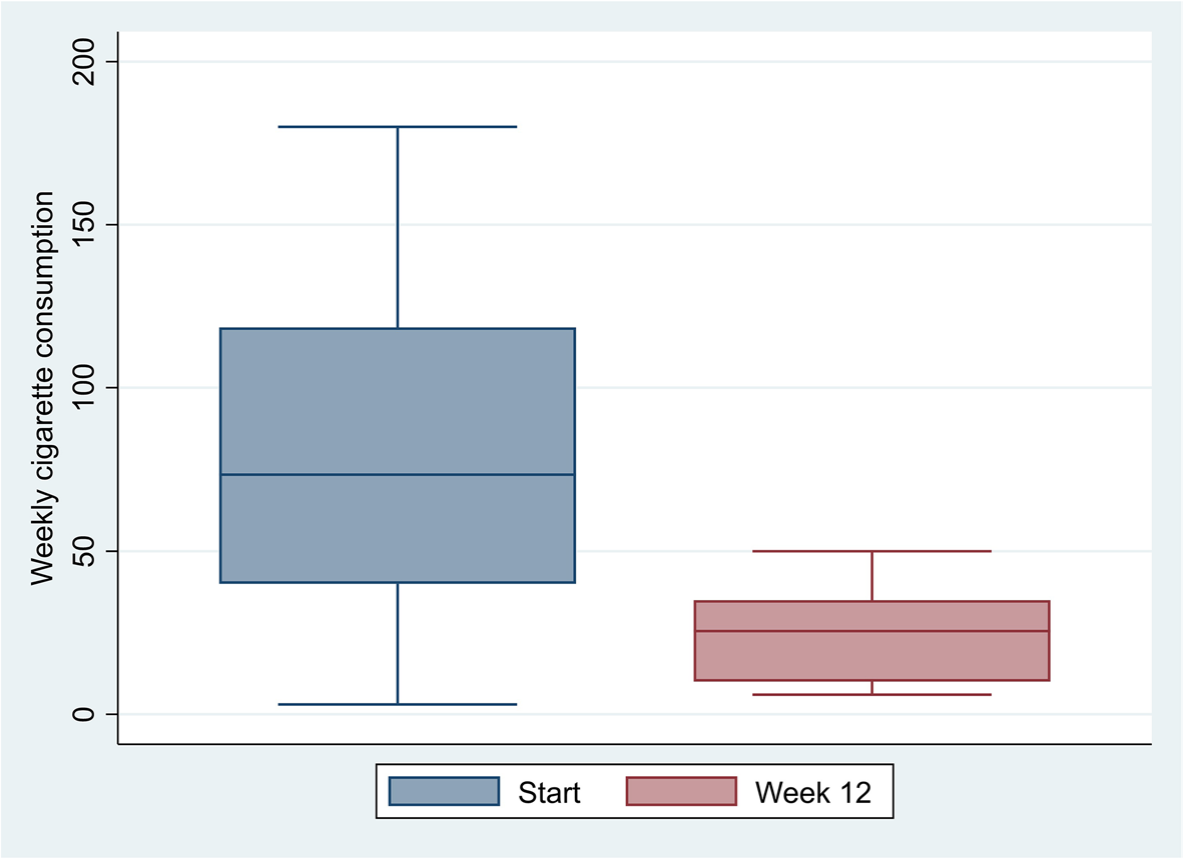
Changes in weekly cigarette consumption for completers of the trial (*n* = 10)

One-third of the participants were women (Table 2). At the start of the intervention, thirteen participants aimed to quit smoking, whereas twelve reported a reduction in cigarette smoking as their goal. The participants’ choices of primary medication to assist smoking cessation are shown in Table 3. Ten participants (40%) completed the full 12 weeks of the trial. There were only small differences in baseline characteristics between participants who completed the trial and those who did not, apart from reporting of mental health problems. At baseline 70% of completers reported SCL-10 scores ≥ 1.85 compared to 47% among non-completers (Additional File 5). The median duration of receiving active intervention was nine weeks (Fig. 2). At the start of the intervention, participants (*n* = 10) who completed the 12 weeks of the trial reported a median of 70 cigarettes per week (range 31–175), compared to a median of 95 (range 3–180) cigarettes among the fifteen participants who did not complete the trial. Among completers, the median reported weekly cigarette consumption in the last week of follow-up was 26 (range 6–50) cigarettes (Fig. 3). Similarly, the carbon monoxide levels were measured to a median of 14 parts per million (ppm) (range 7–22) and 12 ppm (range 5–17) at the start and end of the trial, respectively. Among the trial completers thirty percent scored ≥ 1.85 in the SCL-10 at the end of the trial. SCL-10 values were missing for three participants at the end of the trial. One participant reported smoking zero cigarettes in week four but did not attend further study visits, including the evaluation in week 12. Another participant continued with the intervention until he relapsed into substance use halfway through and subsequently missed appointments. The participant was interviewed during inpatient withdrawal treatment.

**Table 2:** Characteristics of the participants (*n* = 25)

Age, median (range)	49 (33–65)
Women/men	8/17
OAT medication, n (%)	
*Methadone*	8 (32)
*Buprenorphine and others*^*a*^	17 (68)
Time receiving OAT n (%)	
* 0–5 years*	4 (16)
≥ *6 years*	16 (64)
Education	
*Not completed 10 years of education*	0
*Completed 10 years of education*	16 (64)
*High school or higher*	8 (32)
Debt difficulties^b^, n (%)	13 (52)
Stable housing conditions, n (%)	25 (100)
Living alone	11 (44)
Debut age, median (range)	
*Alcohol*	13 (7–16)
*Cannabis*	15 (7–26)
*Stimulants*	24 (14–31)
*Opioids*	20 (12–50)
*Benzodiazepines*	16 (12–30)
*Tobacco*	13 (7–21)
Injected drugs past 6 months, n (%)	7 (28)
Obstructive pulmonary disease,^c^ n (%)	8 (32)
Fagerström nicotine dependence score median (range)	6 (1;8)
SCL-10 ≥ 1.85 n (%)^d^	14 (56)

^a^Other OAT medication such as long-acting morphine formulations

^b^not able to pay off legal or illegal debt

^c^FEV1/FVC ratio after inhalation of salbutamol < 70% [32]

^d^Scores ≥ 1.85 indicate mental health problems in the general population [28]

**Table 3:** Choice of medication at the start of the trial in addition to short motivational counselling

*Primary medication, n (%)*		*As-needed medication, n (%)*	
Varenicline	12 (48)	Nicotine lozenges	9 (36)
Nicotine patch	9 (36)	Nicotine chewing gum	2 (8)
Nicotine lozenges	4 (16)	None	14 (56)
*Total*	25 (100)	*Total*	25 (100)

Nine interviews involving ten participants were completed; two participants living together wanted to conduct the interview together. The mean interview duration was 18 min. The following themes were identified: usefulness of the intervention, experienced support from staff, acceptability of intervention, usefulness of medication for nicotine withdrawal symptoms, and reasons for discontinuation of the intervention.

### Usefulness of the intervention

Most participants agreed that the intervention helped them reduce the number of cigarettes smoked or quit smoking. They reported that the medication reduced cigarette cravings and even helped them reduce cannabis smoking. Lozenges helped alleviate nicotine cravings when they were taking public transportation or in other places where they could not smoke. However, participants perceived the patches as the most effective, and one participant described how the patch alleviated the cravings:

*“I had no cigarette cravings in the morning, and those are [usually] the worst…"*

Some participants expressed disappointment, not having managed to quit smoking during the intervention period. Many participants indicated that the three months of the intervention were too short, and they would have preferred that the intervention be extended and tailored to each participant's needs. Another participant summarised the thoughts about the intervention as follows:

*"I'm a little disappointed that I didn't manage to quit. It bothers me. But I'm happy I managed to halve it, though. I used to smoke 30–40 cigarettes a day."*

### Experienced support from staff

All participants stressed that support from the nurses providing follow-up was an essential element of the intervention. They reported that it helped them follow the intervention plan and motivated them to attend the weekly appointments. Attending the weekly appointments was feasible for the participants. The conversations with the nurses were encouraging, and they liked having someone to talk to.

*«…It was essential, it was. If he [nurse] hadn't been there I' m sure I wouldn't even have tried to stop smoking."* (Male participant).

### Acceptability of intervention

The participants reacted positively when they learned that the OAT clinics would offer them a smoking cessation intervention. Participants had not expected help with smoking cessation at OAT clinics. Most highlighted receiving smoking cessation medication for free and having an individual choice between products, as exemplified by the following quotation from a participant:

*"… because I have been addicted to pills. Therefore, if I were to swap the smoke for tablets [varenicline], it is like… I would not risk it [triggering addiction to pills]. I chose nicotine patches, nicotine gums and lozenges … The patches were the most helpful."*

Another participant explained how the plan for the daily medication and the checklist for smoking cessation was helpful:

*“Well, you got to take it [the smoking cessation medications] home with you, and then you had the plan and the checklist, so you could see that it [the number of cigarettes] went down."*

### Reasons for discontinuation

Three participants talked about their reasons for stopping the intervention. Two participants experienced strong side effects from the nicotine patches: palpitations and tachycardia aggravated the existing heart problems, and the heartburn worsened. One participant switched to varenicline but experienced the same side effects. The other continued using only lozenges. One participant expressed his disappointment in not managing to quit smoking:

*“I’m disappointed, I could not complete [because of side-effects] and had to give it up before I was done, right? [I] just had to stop [using smoking cessation medication]."*

## Discussion

This pilot study examined the feasibility of integrating smoking cessation therapy into the treatment program of OAT outpatient clinics. The findings indicate that this is an acceptable and feasible method of providing a smoking intervention for patients at OAT clinics. The study further indicated that the participants generally had a positive experience, and many substantially reduced the number of cigarettes smoked. However, only forty percent completed the full 12 weeks of the intervention. Participants stressed that support from staff was a vital element of the intervention.

Despite a considerable reduction in the number of cigarettes smoked and a median period of intervention of nine weeks, only one participant reported stopping smoking. The low smoking cessation rate indicated a reduced feasibility of the intervention tested for providing smoking cessation. Simultaneously, the results indicate that a harm reduction strategy aiming at smoking reduction was feasible with the intervention tested. The median value of the carbon monoxide measurements was reduced at the end of the trial compared to baseline for the participants who completed the trial. This corroborates the participants’ self-report and strengthens the assumption of the interventions’ feasibility in reducing smoking among individuals receiving OAT. There was little chance in mental health scores for participants completing the trial. This observation may be attributable to the relatively low rate of smoking cessation, given that previous studies have documented decreases in mental health scores among individuals who successfully discontinued smoking [33, 34].

The qualitative analysis indicated that most interviewees recommended longer and more individually adjusted durations of the provision of smoking cessation medications. Two randomised controlled trials among patients receiving buprenorphine and methadone for opioid dependence found low adherence to the extended smoking cessation intervention provided for up to six months and low cessation rates at six months [35, 36]. Yet, future trials should test if an individualized duration of an integrated smoking cessation intervention would improve cessation outcomes.

Education level, perceived stress, and depressive and anxiety symptoms have been linked to poor adherence to smoking cessation interventions [37, 38]. In our sample, two-thirds of the participants reported ten or fewer years of education. In addition, half of the participants had debt difficulties and low levels of psychological well-being, indicating that these factors probably impacted the adherence in our study. However, the participants who completed the trial reported more mental health issues compared to non-completers indicating that mental health issues had less influence on adherence in our trial. Another aspect likely contributing to the 40% completion rate in our trial was perhaps the disruption of follow-up during summer vacation, suggesting that care must be taken in future trials to ensure continuous clinician follow-up. However retention rates in feasibility studies among individuals receiving OAT are generally low. For example, one study reported a retention rate of 24% for combined counselling and nicotine replacement among individuals receiving methadone [12]. Another study reported retention rates ranging from 38 to 57% for varenicline used for smoking cessation among individuals receiving buprenorphine for opioid dependence [39]. In contrast, a study involving electronic cigarettes for individuals receiving buprenorphine/naloxone showed a higher retention rate of 71% [12, 40]. Social support and attending more healthcare visits have increased adherence to smoking cessation [37, 38]. The provision of free medications and weekly nurse support as part of our intervention may not be feasible in resource-limited settings. Involving community health workers could help address this challenge. Among persons with serious mental health diagnoses, the additional support of community health workers increased tobacco abstinence [41, 42]. A qualitative study among OAT patients who smoked found that many planned to stop smoking, but few put their plans into action [16]. Thus, interventions improving accessibility to start a smoking cessation attempt involving both community workers and specialized clinics appear essential. In addition, integrating a smoking cessation intervention in outpatient OAT clinics probably has improved healthcare providers’ low expectations regarding smoking cessation among patients [16, 43]. Offering such interventions to patients demonstrates hope, and when patients perceive the interventions as meaningful, social support in the clinic is also strengthened [44].

Participants expressed unanimously positive attitudes towards receiving smoking cessation treatment at their OAT clinic. Another pilot study revealed that retention in smoking cessation treatment increased with the duration of treatment for opioid dependency [45]. In our study, two-thirds of the participants received OAT for six years or more. A systematic review of concurrent smoking cessation and treatment for other addictions concluded that smoking cessation therapy did not affect abstinence from alcohol or other drugs [26]. At the same time, low medication adherence was found to be a specific challenge facing smokers with opioid dependence [9]. Integration of treatment for concomitant diseases at OAT clinics effectively increases treatment rates for diseases such as hepatitis C virus infection [46]. The potential effect size of such interventions on smoking cessation should be tested in a sufficiently powered randomised controlled trial.

Some participants in our study reported side effects from smoking cessation medication as a reason for discontinuation. Still, none of the interviewees reported relapse to drug use as a result of participation in the trial. Persons in treatment for substance dependencies reported concerns regarding increased craving or fear of relapse from other substances when quitting smoking [47, 48]. These concerns were in part also shared by staff and could impact the provision of smoking cessation therapy [16, 49]. Fear of relapse to drug use seems unfounded as several studies and reviews have found that smoking cessation does not impact substance use and, instead, in some cases, might promote abstinence from substance use [26, 50, 51]. We used a small incentive (equivalent of approximately 20 Euros) to compensate for the time used for participation. This could give stronger incentives for those with difficult economy to participate. However, harmful effects of participation seemed unlikely.

This study had several limitations. The study did not include a control group or use a randomization design. Participants were recruited purposively, in which research nurses approached patients at the OAT clinics whom they knew from previous health assessments and expected could complete the study. The participants in our study had a similar mean age (49 vs 48 years) and proportion of methadone users (30% vs 33%) when comparing to the national Norwegian population of persons receiving OAT [21]. Our sample had a larger proportion of females (47% vs 30%), and all participants reported a stable housing situation compared to 79% in the national sample [21]. Our sample was similar to a larger sample recruited from the same OAT population as our study, regarding the mean (standard deviation) time receiving OAT, which was 8 [6] years, whereas in our sample, two thirds had received OAT for more than 6 years [52]. In our sample, all participants had completed primary school, compared to 95% of the larger sample from the same population [52]. Thus, the sample in our study is did not capture the diversity of the OAT population. The feasibility of this study could not be evaluated for individuals with unstable housing conditions and less than ten years of education, indicating that our sampling strategy placed too much emphasis on the expectation of completing the study when recruiting participants. The purpose of this study was not to test the effectiveness of the intervention but rather to test the acceptability among participants and the feasibility of integration into the standard programme of OAT clinics. Given the specific aims, we used the concept of information power and reported sample sizes in other qualitative feasibility studies to guide our sample sizes [20, 22]. However, the sample size estimations seem to have underestimated the likelihood of dropout, specifically in the summer months, potentially leading to a loss of information. To address this, we have included a supplementary table showing the distribution of potential confounders among those who completed the trial and those who did not. The timing of the intervention, coinciding primary summer vacation month of July, represents a limitation from an efficacy perspective. The vacation resulted in insufficient continuity between clinicians, including the nurses providing intervention follow-up and evaluation. Consequently, several participants did not receive follow-up according to the protocol. Other staff at the OAT clinics handed out the smoking cessation products to some degree, but the weekly registrations and micro motivational talks were generally left out during this period. Simultaneously, this also made it more transferable to typical clinical contexts, which could be important when assessing effectiveness.

## Conclusions

This study indicated that patients receiving OAT welcomed smoking cessation therapy provided at the OAT clinics. Most patients found it acceptable, and most participants who completed the trial managed to reduce the number of cigarettes smoked to less than half. However, the intervention assessed resulted in low quit rates, indicating less feasibility for providing smoking cessation. Simultaneously, the reported reduction in cigarettes smoked suggests that the intervention could be a feasible component of a harm reduction strategy. Several participants argued that the quit rates following the smoking cessation intervention could have been higher if the smoking cessation intervention had been provided for a longer period. Integrating smoking cessation into the daily program of the OAT clinics was also experienced as feasible within the involved clinics. Our study also showed that disruptions in follow-up need to be planned for to increase participant retention. Future trials should also plan for more structured training and involvement of clinicians unrelated to the research group. By involving more clinicians, continuity of care can likely improve over time. An adequately powered randomised controlled trial is needed to evaluate the effect of such integrated smoking treatment, allowing for the testing of a cessation and harm reduction strategy.

## Supplementary Information

Additional file 1: CONSORT 2010 checklist of information to include when reporting a pilot or feasibility trial*.

Additional file 2: Consolidated criteria for reporting qualitative research (COREQ)(1).

Additional file 3: Consolidated criteria for reporting qualitative research (COREQ)(1).

Additional file 4: Main steps of qualitative analysis according to systematic text condensation (1). Figure is adapted from (2) under the terms of a Creative Commons Attribution License (CC BY).

Additional file 5: Distribution of potential confounders among participants at baseline (*n* = 25).

## Data Availability

The datasets generated and/or analysed during the current study are not publicly available because they contain personal identifiable data, but they are available from the corresponding author on reasonable request.

## Abbreviations

OAT: Opioid agonist therapy
NRT: Nicotine replacement therapy
SCL-10: Hopkins Symptom Checklist, ten items
