# Supplementary material for: Multimethod feasibility evaluation of smoking cessation intervention for patients receiving opioid agonist therapy
Source: Pilot Feasibility Stud. 2025 Oct 31;11:128. doi: 10.1186/s40814-025-01717-2 (PMC12577004; doi:10.1186/s40814-025-01717-2)
Supplement: Supplementary file 2 — Additional file 2: Consolidated criteria for reporting qualitative research (COREQ)(1). [file 40814_2025_1717_MOESM2_ESM.docx]

l

**Consolidated criteria for reporting qualitative research (COREQ)(1):**

| No. | Item | Guide questions/ descriptions |  |
| --- | --- | --- | --- |
| **Domain 1: Research team and reflexivity** | | | |
| 1. | Interviewer/facilitator | Which author/s conducted the interview or focus group? | The first author (KTDF) |
| 2. | Credentials | What were the researcher’s credentials? *E.g. PhD, MD* | KLTD: MD and Phd-candidate, TM: MD and Phd, SELC: M.sc. and PhD, EF: M.Sc. and Phd-candidate, JHV: MD and Phd, JTD M.Sc. and Phd-candidate, TGL: MD and PhD LTF: MD and PhD |
| 3. | Occupation | What was their occupation at the time of the study? | All researcher were employed by the Bergen addiction research group at Haukeland university hospital in Bergen, Norway |
| 4. | Gender | Was the researcher male or female? | Both |
| 5. | Experience and training | What experience or training did the researcher have? | The senior researchers (SELC; LTF AND TGL) have previous experience in qualitative research. The first author has puplished a previous qualitative paper. |
| 6. | Relationship established | Was a relationship established prior to study commencement? | Research nurses knew participants from yearly health assessments. The researcher conducting the interviews met the participants for the first time when the interviews were conducted. |
| 7. | Participant knowledge of the interviewer | What did the participants know about the researcher*? e.g. personal goals, reasons for doing the research* | Participants were informed that the research was part of a larger study on smoking cessation (ATLAS4LAR) and that the first author was working part-time as a researcher and a clinician (but not in the department, from which the participants were recruited. |
| 8. | Interviewer characteristics | What characteristics were reported about the interviewer/facilitator? *e.g. Bias, assumptions, reasons and interests in the research topic* | No characteristics were reported |
| **Domain 2: study design** | | | |
| 9. | Methodological orientation and Theory | What methodological orientation was stated to underpin the study? *e.g. grounded theory, discourse analysis, ethnography, phenomenology, content analysis* | We used systematic text condensation (2) to guide the design and analysis of the study. |
| 10. | Sampling | How were participants selected? *e.g. purposive, convenience, consecutive, snowball* | The sampling was purposive. See pages 6-7 for further information. |
| 11. | Method of approach | How were participants approached? *e.g. face-to-face, telephone, mail, email* | Participants were approached by telephone and face to face. |
| 12. | Sample size | How many participants were in the study? | 25 |
| 13. | Non-participation | How many people refused to participate or dropped out? Reasons? | 15  One patient was unavailable, the 4 other were offered participation in interviews, but declined or did not show up for the interview. |
| 14. | Setting of data collection | Where was the data collected? *e.g. home, clinic, workplace* | At the patients’ local OAT- clinic |
| 15. | Presence of non-participants | Was anyone else present besides the participants and researchers? | No |
| 16. | Description of sample | What are the important characteristics of the sample? *e.g. demographic data, date* | See table 2, page 11 |
| 17. | Interview guide | Were questions, prompts, guides provided by the authors? Was it pilot tested? | The interview guide was semi structured with open end questions. The interview guide was not piloted but developed together with user representatives. |
| 18. | Repeat interviews | Were repeat interviews carried out? If yes, how many? | None were repeated |
| 19. | Audio/visual recording | Did the research use audio or visual recording to collect the data? | Interviews were audio-recorded |
| 20. | Field notes | Were field notes made during and/or after the interview or focus group? | No |
| 21. | Duration | What was the duration of the interviews or focus group? | Mean duration was 18 minutes |
| 22. | Data saturation | Was data saturation discussed? | The authors discussed saturation during the analysis process. |
| 23. | Transcripts returned | Were transcripts returned to participants for comment and/or correction? | No |
| **Domain 3: analysis and findings** | | | |
| 24. | Number of data coders | How many data coders coded the data? | The first author proposed the preliminary themes. Preliminary themes wer discussed with EF, SELC and TM before the codes were developed. The first author extracted the meaning units. |
| 25. | Description of the coding tree | Did authors provide a description of the coding tree? | Additional file 3 |
| 26. | Derivation of themes | Were themes identified in advance or derived from the data? | Themes were derived from the data. |
| 27. | Software | What software, if applicable, was used to manage the data? | The NVivio software was used to manage the data. |
| 28. | Participant checking | Did participants provide feedback on the findings? | No |
| 29. | Quotations presented | Were participant quotations presented to illustrate the themes / findings? Was each quotation identified? *e.g. participant number* | presented in the manuscript. Each participant was given a pseudonym which was used to identify the quotes |
| 30. | Data and findings consistent | Was there consistency between the data presented and the findings? | There is consistency between presented data and the findings (see pages 10-14) |
| 31. | Clarity of major themes | Were major themes clearly presented in the findings? | The major themes are clearly presented with subheadings |
| 32. | Clarity of minor themesa | Is there a description of diverse cases or discussion of minor themes? | Diverse cases and minor themes are discussed in the paper |

1. Tong A, Sainsbury P, Craig J. Consolidated criteria for reporting qualitative research (COREQ): a 32-item checklist for interviews and focus groups. Int J Qual Health Care. 2007;19(6):349-57.

2. Malterud K. Systematic text condensation: a strategy for qualitative analysis. Scand J Public Health. 2012;40(8):795-805.
