# Supplementary material for: Multimethod feasibility evaluation of smoking cessation intervention for patients receiving opioid agonist therapy
Source: Pilot Feasibility Stud. 2025 Oct 31;11:128. doi: 10.1186/s40814-025-01717-2 (PMC12577004; doi:10.1186/s40814-025-01717-2)
Supplement: Supplementary file 3 — Additional file 3: Consolidated criteria for reporting qualitative research (COREQ)(1). [file 40814_2025_1717_MOESM3_ESM.docx]

# Interview guide evaluation of the pilot smoking cessation intervention

*( Translated from Norwegian)*

We would like to ask some questions about your experiences of having completed the smoking cessation intervention, but first some questions about your thoughts before you started this project.

- What expectations did you have for participating in the project?
- What was the motivation for participating?
- What are you left with after participating in the trial (main impression)?
  - was there anything you found useful/positive about participating?
  - was there anything you found negative/problematic?
  - Did you participate the full 12 weeks - why/why not?

*If necessary, follow up on themes theme from initial response:*

- What do you think about using medications (varenicline, nicotine patch, nicotine gum etc.) to reduce or stop smoking?
- To what extent do you think it is achievable to reduce/stop smoking?
- How are your smoking habits now (ask if not already answered)?
- Did you achieve the goals set at the start of the trial?
- What do you think about the duration treatment (too short/long/appropriate time, desire for extension)?

*For participants still smoking:*

- Have you made any changes to your smoking habits (ask if not already answered)

*All participants:*

- Has taking part in the project changed the way you think about your own health?
  - Has participating in the project led to you making changes in your everyday life? If so, what changes?
  - With the experience you have from the project you participated in, what do you think about your exercise habits /smoking habits/diet in the future?
- Going forward, how do you think it will be possible to maintain the changes you have achieved?
- Are there any changes/possible other changes that you think will be relevant for you in the future? (What do you need to maintain this?)
- Is there something you think should have been done differently/something you miss in the study as it was conducted?
- How did you experience the length of the project (ok/too short/long, other?)
- How did you experience the commitment of the project's instructor? What significance did it have for you and your participation?
- Something you would like to add, something on your mind that I haven't asked?
