# Supplementary material for: Multimethod feasibility evaluation of smoking cessation intervention for patients receiving opioid agonist therapy
Source: Pilot Feasibility Stud. 2025 Oct 31;11:128. doi: 10.1186/s40814-025-01717-2 (PMC12577004; doi:10.1186/s40814-025-01717-2)
Supplement: Supplementary file 4 — Additional file 4: Main steps of qualitative analysis according to systematic text condensation (1). Figure is adapted from (2) under the terms of a Creative Commons Attribution License (CC BY). [file 40814_2025_1717_MOESM4_ESM.docx]

 **Additional File 4.** Main steps of qualitative analysis according to systematic text condensation (1). Figure is adapted from (2) under the terms of a Creative Commons Attribution License (CC BY).

**References**

1. Malterud K. Systematic text condensation: a strategy for qualitative analysis. Scand J Public Health. 2012;40(8):795–805.

2. Druckrey-Fiskaaen KT, Furulund E, Madebo T, Carlsen SL, Fadnes LT, Lid TG. A qualitative study on people with opioid use disorders' perspectives on smoking and smoking cessation interventions. Front Psychiatry. 2023;14:1185338.
