# Supplementary material for: Multimethod feasibility evaluation of smoking cessation intervention for patients receiving opioid agonist therapy
Source: Pilot Feasibility Stud. 2025 Oct 31;11:128. doi: 10.1186/s40814-025-01717-2 (PMC12577004; doi:10.1186/s40814-025-01717-2)
Supplement: Supplementary file 5 — Additional file 5: Distribution of potential confounders among participants at baseline (n = 25). [file 40814_2025_1717_MOESM5_ESM.docx]

**Additional File 5: Distribution of potential confounders among participants at baseline (n=25)**

| **Variable** | **Completer** | **Non-completer** |
| --- | --- | --- |
| SCL-10 ≥ 1.85 n (%)^1^ | 7 (70) | 7 (47) |
| Injected drugs past 6 months, n (%) | 2 (20) | 5 (36) |
| Female, n (%) | 3 (30) | 4 (29) |
| OAT-medication, n (%) |  |  |
| Buprenorphine and others^2^ | 6 (60) | 11 (79) |
| Methadone | 4 (40) | 3 (21) |
| Nicotine dependence score (Fagerström), median (Range) | 6 (5-8) | 5 (2-8) |

^1^Scores ≥ 1.85 indicate mental health problems in the general population (1)

^2^Other OAT medication such as long-acting morphine formulations

1. Strand BH, Dalgard OS, Tambs K, Rognerud M. Measuring the mental health status of the Norwegian population: a comparison of the instruments SCL-25, SCL-10, SCL-5 and MHI-5 (SF-36). Nord J Psychiatry. 2003;57(2):113–8.
